# Selenium-chitosan alleviates the toxic effects of Zearalenone on antioxidant and immune function in mice

**DOI:** 10.3389/fvets.2022.1036104

**Published:** 2022-10-06

**Authors:** Shunyi Qin, Fuze She, Fanghong Zhao, Liuan Li, Fu Chen

**Affiliations:** ^1^Tianjin Key Laboratory of Agricultural Animal Breeding and Healthy Husbandry, College of Animal Science and Veterinary Medicine, Tianjin Agricultural University, Tianjin, China; ^2^College of Veterinary Medicine, Qingdao Agricultural University, Qingdao, China

**Keywords:** selenium-chitosan, Zearalenone, mice, antioxidant function, immune function

## Abstract

This study assessed the protective effects of selenium-chitosan (SC) against antioxidant and immune function-related damage induced by zearalenone (ZEN) in mice. In total, 150 female mice were allotted to five groups for a 30-day study. Control mice were fed a basal diet. Mice in the ZEN, ZEN-Se1, ZEN-Se2 and ZEN-Se3 groups were fed the basal diet supplemented with same dose of ZEN (2 mg/kg) and different doses of SC, 0.0, 0.2, 0.4 and 0.6 mg/kg, respectively (calculated by selenium). After 30 days, the total antioxidant capacity (T-AOC) level, glutathione peroxidase (GSH-Px) activity, total superoxide dismutase (T-SOD) activity and malondialdehyde (MDA) content in plasma and liver, as well as Con A-induced splenocyte proliferation, plasma interleukins concentrations and liver interleukin mRNA expression levels were determined. The plasma and liver GSH-Px activities, liver T-AOC levels, Con A-induced splenocyte proliferation, interleukin (IL) contents and mRNA expression levels in the ZEN group were significantly lower than in the control group (*P* < 0.01 or *P* < 0.05), whereas plasma and liver MDA contents in the ZEN group were significantly higher than in the control group (*P* < 0.01 or *P* < 0.05). Additionally, plasma and liver GSH-Px activities, liver T-AOC levels, Con A-induced splenocyte proliferation, IL-1β, IL-17A, IL-2 and IL-6 contents and mRNA expression levels in ZEN+Se2 and ZEN+Se3 groups were significantly higher than in the ZEN group (*P* < 0.01 or *P* < 0.05), whereas plasma and liver MDA contents in the ZEN+Se2 and ZEN+Se3 groups were significantly lower than in the ZEN group (*P* < 0.01 or *P* < 0.05). The plasma and liver GSH-Px activities, Con A-induced splenocyte proliferation, IL-1β and IL-6 contents, IL-2 and IL-17A mRNA expression levels in the ZEN+Se1 group were also significantly higher than in the ZEN group (*P* < 0.01 or *P* < 0.05), whereas the plasma MDA content in the ZEN+Se1 group was also significantly lower than in the ZEN group (*P* < 0.01). Thus, SC may alleviate antioxidant function-related damage and immunosuppression induced by ZEN in mice.

## Introduction

Zearalenone (ZEN) is a mycotoxin produced by Fusarium fungi that widely exist in various grain crops and feedstuff (1, 2). The mainly toxic effect of ZEN is reproductive toxicity, and it also causes hepatotoxicity, nephrotoxicity, immunotoxicity, cytotoxicity and genotoxicity (3, 4). The long-term consumption of ZEN-contaminated feed causes declines in growth performance, immunodepression and reproductive dysfunction in livestock and poultry (5–7). More importantly, when animals eat feed contaminated with ZEN, it remains in the resulting animal products, such as meat, milk and eggs, which can harm human health (2, 8). Therefore, how to relieve the toxic effects of ZEN on humans and animals has become a current research hot spot.

Oxidative damage is a main pathway of ZEN toxicity (9). The oxidative damage mechanism of ZEN to cells is caused by the lipid peroxidation of polyunsaturated fatty acids in their membranes and the formation of lipid peroxide. Lipid peroxidation can eventually give rise to the formation of a variety of lipid decomposition products, some of which alter cell metabolism and cause dysfunction; meanwhile, oxygen radicals induced by ZEN can also cause cell damage through lipid hydrogen peroxide decomposition products (9, 10). Many substances with antioxidant properties have antagonistic effects on ZEN toxicity. For example, vitamin C alleviates oxidative stress induced by ZEN in piglet livers (11); and proanthocyanidins inhibit the apoptosis of mouse intestinal epithelial cells induced by ZEN (12). In recent years, selenium has been shown to alleviate the acute (13) and chronic (14) toxicity of ZEN in mice by improving the antioxidant capacity and inhibiting apoptosis (14, 15). Similarly, Xiao et al. (16) found that selenium protects chicken spleen lymphocyte from ZEN-induced oxidative stress and apoptosis.

Selenium-chitosan (SC) is an organic compound of selenium and chitosan that can simultaneously play the dual roles of organic selenium and chitosan (17). SC improves the performances of animals, regulates the immune state of the body, prevents oxidative stress, inhibits apoptosis and reduces blood sugar and blood lipid levels (17, 18). It also protects against plasma TNF-α and IL-18 changes caused by ZEN in mice (19). However, it is still unclear whether SC can protect against the decreased antioxidant capacity and immune function damage induced by ZEN in mice. In addition, the protective mechanism needs to be elucidated. Therefore, the aim of this study was to investigate whether SC can reduce the toxic effects of ZEN in female mice.

## Materials and methods

### Animals and diets

In total, 150, 3-week-old female Kunming mice (Animal Center, Chinese Academy of Military Medical Sciences, China) were allotted to five treatments of 30 mice each for a 30-day study. Each treatment was replicated six times, with six cages of five mice per cage in each replicate. Mice in the control group were fed a basal diet. Mice in the ZEN, ZEN-Se1, ZEN-Se2 and ZEN-Se3 groups were fed the basal diet supplemented with same dose of ZEN (2 mg/kg) and different doses of SC, 0.0, 0.2, 0.4 and 0.6 mg/kg, respectively (calculated by selenium). Routine feeding and management throughout the trial.

### Sampling and processing

At the end of experiment, three mice from each replicate were selected and harvested under halothane anesthesia. Blood and liver samples were routinely collected for the determination of total antioxidant capacity (T-AOC) level, glutathione peroxidase (GSH-Px) activity, total superoxide dismutase (T-SOD) activity and malondialdehyde (MDA) content. And spleen samples were routinely collected for the determination of the mitogen-induced splenocyte proliferation.

### Laboratory assay

#### Measurement of antioxidant function in plasma and liver

Plasma and liver homogenate suspension samples were collected. T-AOC levels, GSH-Px activities, T-SOD activities and MDA contents in plasma and in liver were determined using the appropriate kits (Nanjing Jiancheng Bioengineering Institute, China). Each measurement was performed in accordance with the manufacturer's instructions, and all the samples were tested in duplicate.

#### Measurement of mitogen-induced splenocyte proliferation

The mitogen-induced splenocyte proliferation was determined in accordance with Qin et al. (17). The stimulate index (SI) was calculated using the following equation:


$$\begin{array}{l} \text{SI}=\mathrm{ConA}{+}_{\left(\text{OD}570\right)}/\mathrm{ConA}{-}_{\left(\text{OD}570\right)} \end{array}$$

#### Measurement of plasma interleukins contents

Plasma interleukin (IL)-1β, IL-17A, IL-2 and IL-6 contents were determined using the appropriate kit (Jiangsu Meimian Industrial Co., Ltd, China). Each measurement was performed in accordance with the manufacturer's instructions, and all the samples were tested in duplicate.

#### Measurement of liver interleukins mRNA expressions

TRIzol reagent (Invitrogen, UK) was used to extract the total RNA of each liver sample. Then, the purity and content of the total RNA were determined by measuring the absorbance ratio of 260/280 nm. The total RNA was reverse transcribed to cDNA using a First-Strand cDNA Synthesis Kit (Genecopoeia, USA).

A CFX96 Touch real-time PCR system (Bio-Rad, USA) and the SYBR Green qPCR mix 2.0 Kit (Genecopoeia) were used to conduct real-time PCR. Relative mRNA expression levels of interleukins were determined using the 2^−ΔΔCt^ method. The primers, which were designed and synthesized by Beijing Sangon Biotech Co., Ltd, are listed in Table 1. The PCR reaction conditions were as follows: initial denaturation at 95°C for 6 min; 40 cycles of denaturation at 95°C for 20 s, annealing at 56°C for 20 s, and extension at 72°C for 30 s; followed by of final extension at 72°C for 5 min.

**Table 1:** The PCR primer sequences for amplifying GADPH, IL-1β, IL-17A, IL-2 and IL-6.

**Genes**	**Product length**	**Primer sequence**	**Genebank access number**
GADPH	126bp	F:5'-TGATGGGTGTGAACCACGAG-3' R:5'-GCCCTTCCACAATGCCAAAG-3'	NM_008084.3
IL-1β	89bp	F:5'-GCAACTGTTCCTGAACTCAACT-3' R:5'-ATCTTTTGGGGTCCGTCAACTCC-3'	NM_008361.3
IL-17A	117bp	F:5'-GGAAAGCTGGACCACCACA-3' R:5'-CACACCCACCAGCATCTTCTC-3'	NM_010552.3
IL-2	96bp	F:5'-AACTGTGGTGGACTTTCTGAG-3' R:5'-ATGTGTTGTAAGCAGGAGGTAC-3'	NM_008366.3
IL-6	142bp	F:5'-CAACGATGATGCACTTGCAGA-3' R:5'-CTCCAGGTAGCTATGGTACTCCAGA-3'	NM_031168.1

### Statistical analysis

The results are presented as means ± standard deviation. The cage was defined as the experimental unit for the statistical analyses, and all calculations were generated using cage averages. Significance levels of differences among multiple groups were analyzed using Tamhane's T2 test, one-way analysis of variance and least significant difference methods. SPSS 22.0 software (SPSS Inc., USA) was used for all the statistical analyses. Mean values were considered to be significantly different at *P* < 0.05.

## Results

### Plasma antioxidant function

**Figure 1:**
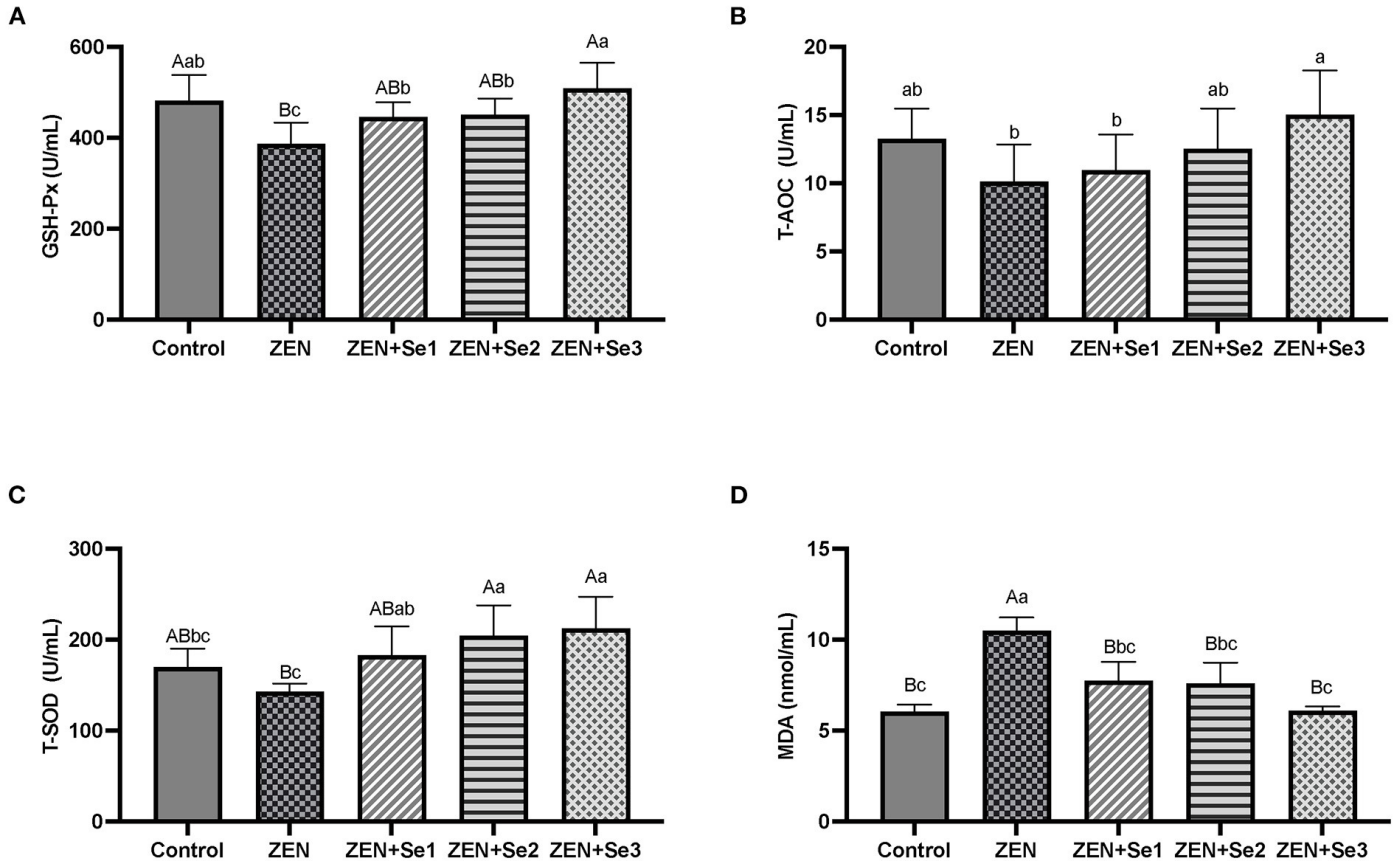
Effects of SC on plasma levels of different parameters induced by ZEN in mice: **(A)** GSH-Px activity; **(B)** T-AOC level; **(C)** T-SOD activity; **(D)** MDA content. Different capital letters (lowercase letters) in the column chart indicate a significant difference at the 0.01 (0.05) level.

As shown in Figures 1A–D, plasma GSH-Px activities of mice in the ZEN group were significantly lower than those of mice in the control group (*P* < 0.01), whereas plasma MDA contents of mice in the ZEN group were significantly greater than those of mice in the control group (*P* < 0.01). Additionally, plasma GSH-Px activities of mice in the ZEN+Se1, ZEN+Se2 and ZEN+Se3 groups were significantly higher than those of mice in the ZEN group (*P* < 0.01 or *P* < 0.05). Plasma MDA contents of mice in the ZEN+Se1, ZEN+Se2 and ZEN+Se3 groups were significantly lower than those of mice in the ZEN group (*P* < 0.01). Plasma GSH-Px activities of mice in the ZEN+Se3 group were higher than those of mice in the ZEN+Se1 and ZEN+Se2 groups (*P* < 0.05).

### Liver antioxidant function

**Figure 2:**
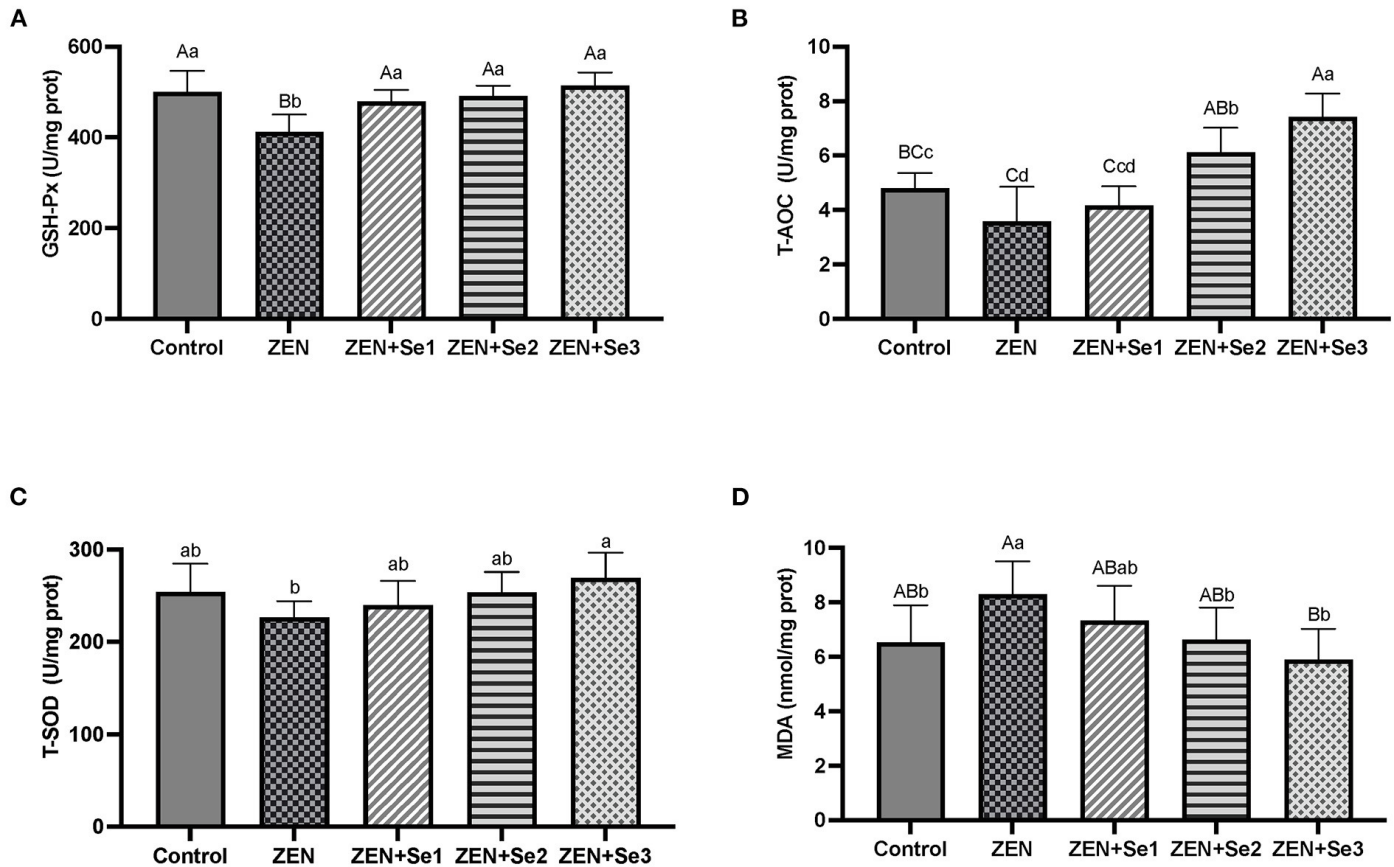
Effects of SC on liver levels of different parameters induced by ZEN in mice: **(A)** GSH-Px activity; **(B)** T-AOC level; **(C)** T-SOD activity; **(D)** MDA content. Different capital letters (lowercase letters) in the column chart indicate a significant difference at the 0.01 (0.05) level.

As shown in Figures 2A–D, liver GSH-Px activities and T-AOC levels of mice in the ZEN group were significantly lower than those of mice in the control group (*P* < 0.01 or *P* < 0.05), whereas liver MDA contents of mice in the ZEN group were significantly greater than those of mice in the control group (*P* < 0.05). Additionally, liver GSH-Px activities of mice in the ZEN+Se1, ZEN+Se2 and ZEN+Se3 groups were significantly higher than those of mice in the ZEN group (*P* < 0.01). Liver T-AOC levels of mice in the ZEN+Se2 and ZEN+Se3 groups were significantly higher than those of mice in the ZEN group (*P* < 0.01), respectively. Liver MDA contents of mice in the ZEN+Se2 and ZEN+Se3 groups were significantly lower than those in the ZEN group (*P* < 0.01 or *P* < 0.05). Liver T-AOC levels of mice in the ZEN+Se3 group were significantly higher than those of mice in the ZEN+Se2 group (*P* < 0.05).

### Mitogen-induced splenocyte proliferation

As shown in Table 2, Con A-induced splenocyte proliferation in the ZEN group was significantly lower than in the control group (*P* < 0.01). In addition, Con A-induced splenocyte proliferation levels in the ZEN+Se1, ZEN+Se2 and ZEN+Se3 groups were significantly higher than in the ZEN group (*P* < 0.01 or *P* < 0.05). No significant differences in Con A-induced splenocyte proliferation were found among the control, ZEN+Se1, ZEN+Se2 and ZEN+Se3 groups.

**Table 2:** Effects of SC on Con A-induced splenocyte proliferation induced by ZEN in mice^*^.

**Groups**	**Con A-_(*OD*570)_**	**Con A+_(*OD*570)_**	**SI**
Control	0.46 ± 0.04	0.85 ± 0.10^Bc^	1.87 ± 0.10^Bb^
ZEN	0.41 ± 0.02	0.68 ± 0.08^Aa^	1.63 ± 0.16^Aa^
ZEN+Se1	0.43 ± 0.04	0.76 ± 0.06^ABab^	1.78 ± 0.11^ABb^
ZEN+Se2	0.45 ± 0.05	0.83 ± 0.09^Bbc^	1.86 ± 0.07^Bb^
ZEN+Se3	0.47 ± 0.05	0.87 ± 0.06^Bc^	1.86 ± 0.11^Bb^

* The different superscript capital letters (lowercase letters) in the same column indicate a significant difference at the 0.01 (0.05) level.

### Plasma interleukins contents

**Figure 3:**
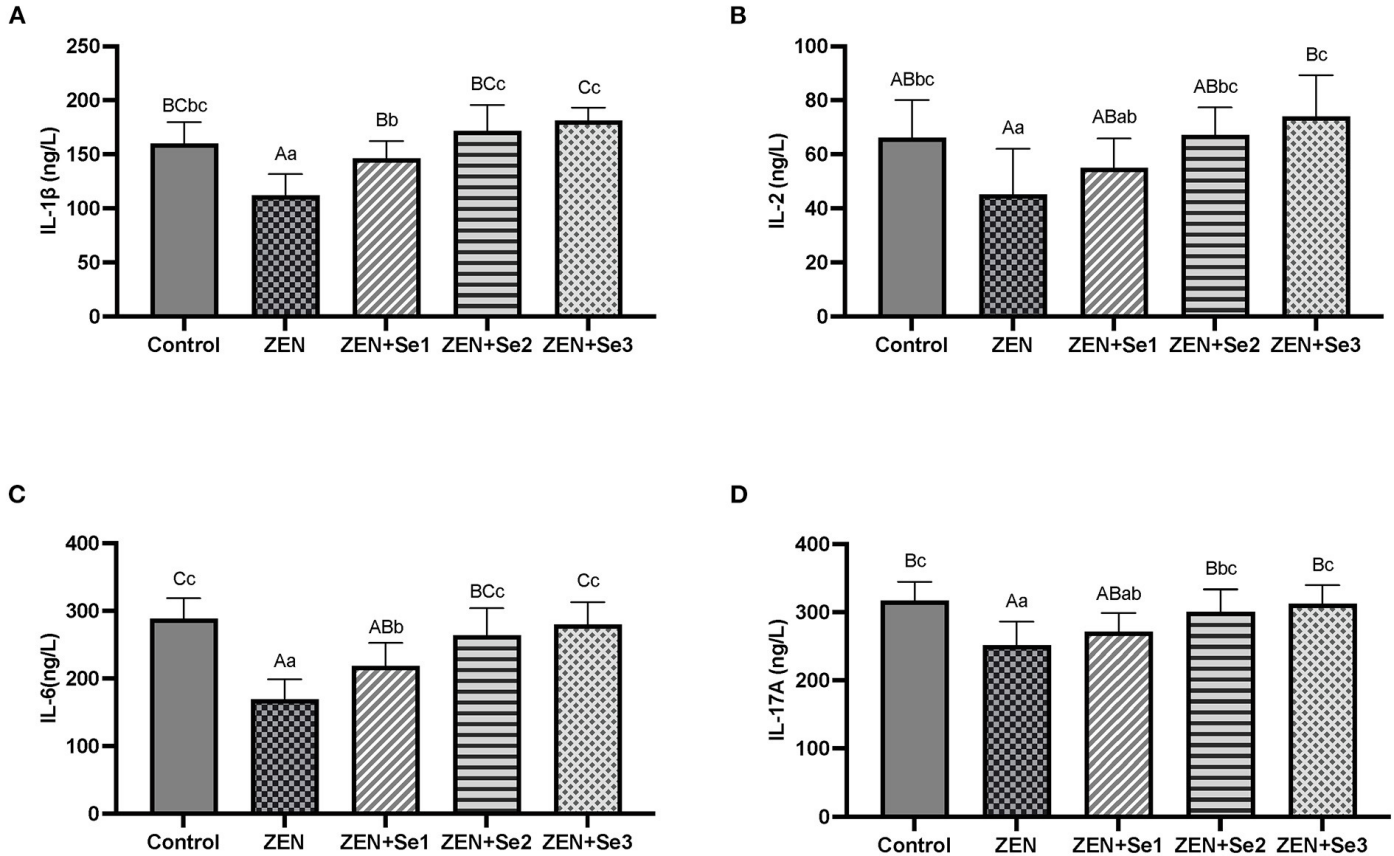
Effects of SC on the plasma contents of ILs induced by ZEN in mice: **(A)** IL-1β; **(B)** IL-2; **(C)** IL-6; **(D)** IL-17A. Different capital letters (lowercase letters) in the column chart indicate a significant difference at the 0.01 (0.05) level.

As shown in Figures 3A–D, the plasma IL-1β, IL-17A, IL-2 and IL-6 contents of mice in the ZEN group were significantly lower than those of mice in the control group (*P* < 0.01 or *P* < 0.05). Plasma IL-1β and IL-6 contents of mice in the ZEN+Se1, ZEN+Se2 and ZEN+Se3 groups were significantly greater than those of mice in ZEN group (*P* < 0.01 or *P* < 0.05). Plasma IL-2 and IL-17A contents of mice in the ZEN+Se2 and ZEN+Se3 groups were significantly greater than those of mice in the ZEN group (*P* < 0.01 or *P* < 0.05). Plasma IL-1β and IL-6 contents of mice in the ZEN+Se2 and ZEN+Se3 groups were significantly greater than those of mice in the ZEN+Se1 group (*P* < 0.01 or *P* < 0.05). Plasma IL-2 and IL-17A contents of mice in the ZEN+Se3 group were greater than those of mice in the ZEN+Se1 group (*P* < 0.05).

### Liver interleukins mRNA expressions

**Figure 4:**
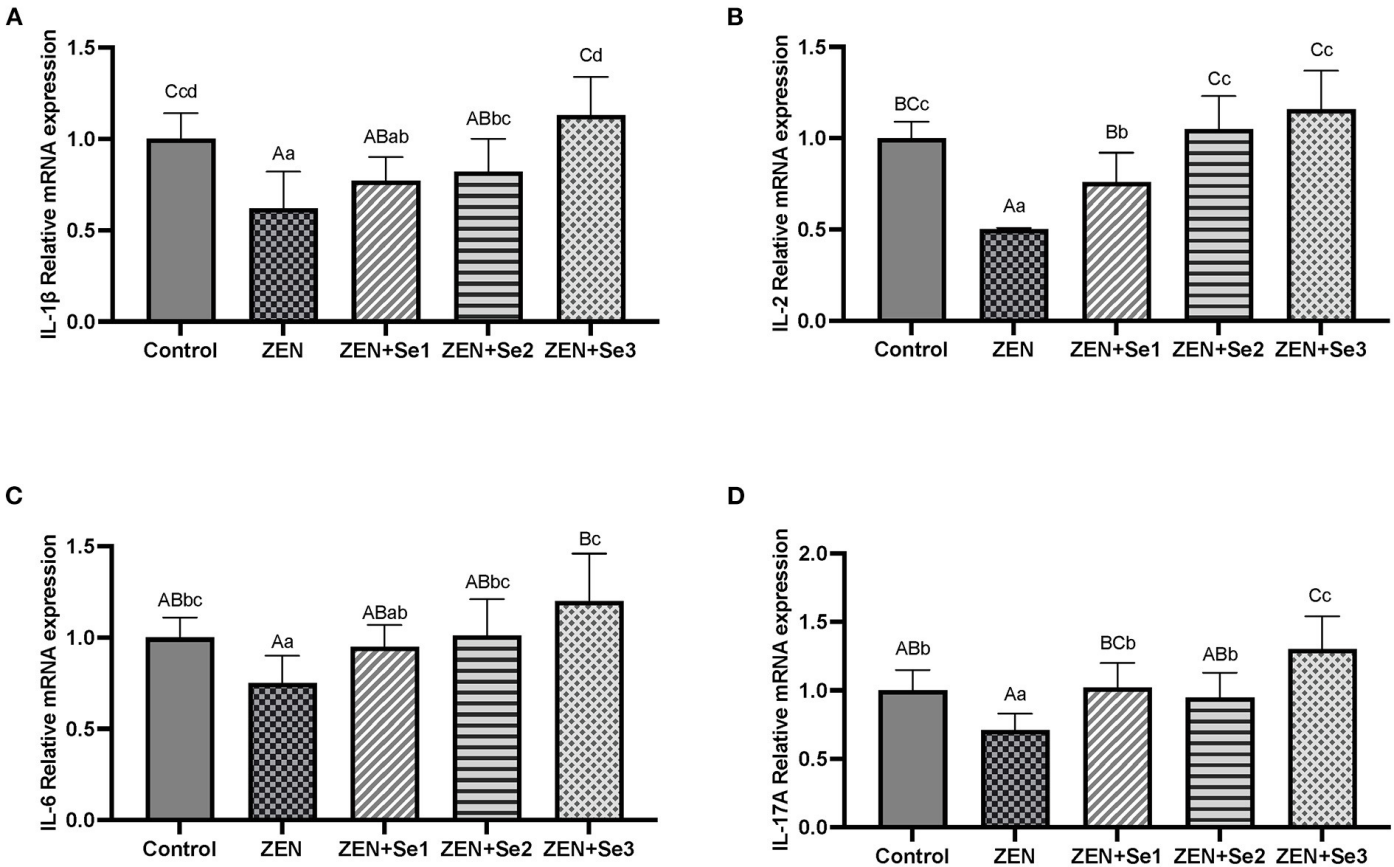
Effects of SC on the liver contents of ILs induced by ZEN in mice: **(A)** IL-1β; **(B)** IL-2; **(C)** IL-6; **(D)** IL-17A. Different capital letters (lowercase letters) in the column chart indicate a significant difference at the 0.01 (0.05) level.

As shown in Figures 4A–D, IL-1β, IL-17A, IL-2 and IL-6 mRNA expression levels of mice in the ZEN group were significantly lower than those of mice in the control group (*P* < 0.01 or *P* < 0.05); whereas IL-1β, IL-17A, IL-2 and IL-6mRNA expression levels of mice in the ZEN+Se2 and ZEN+Se3 groups were significantly higher than those of mice in the ZEN group (*P* < 0.01 or *P* < 0.05). In addition, IL-2 and IL-17A mRNA expression levels of mice in the ZEN+Se1 group were significantly higher than those of mice in the ZEN group (*P* < 0.01).

## Discussion

In recent years, selenium has been shown to have antagonistic effects on the toxicity of ZEN (13, 15, 16). Additionally, a few studies focused on the effects of SC on the toxicity of ZEN. However, it is still unclear whether SC can protect against the reduction in antioxidation capacity and damage to immune functions induced by ZEN. Consequently, this was the focus of our current study.

The T-AOC level, GSH-Px activity, T-SOD activity and MDA content are commonly used to assess antioxidant function. In this study, the level of MDA was used as an indicator of oxidative damage, and the level of T-AOC, as well as the activities of GSH-Px and T-SOD, were used as indicators of antioxidation. Compared with the control group, the plasma GSH-Px activity, liver GSH-Px activity and T-AOC level in the ZEN group decreased, whereas the plasma and liver MDA contents of in the ZEN group increased. Compared with the ZEN group, the plasma and liver GSH-Px activities, as well as the T-AOC levels, in the ZEN+Se2 and ZEN+Se3 groups increased, whereas the plasma and liver MDA contents in the ZEN+Se2 and ZEN+Se3 groups decreased. In addition, compared with the ZEN group, the plasma and liver GSH-Px activities in the ZEN+Se1 group also increased, whereas the plasma MDA content in the ZEN+Se1 group also decreased (Figures 1, 2). The results indicated that ZEN caused damage to antioxidant functions of the liver and blood in mice and that SC could alleviate the damage.

Oxidative damage is a main pathway of ZEN toxicity. It causes lipid peroxidation of unsaturated fatty acids in the body, which produces a variety of lipid decomposition products. The presence of these products triggers metabolic disorders and cellular and tissue dysfunction (9, 10). ZEN can induce tissue cell apoptosis as a result of damage caused by oxidative stress (20). Selenium can alleviate the toxic effects of ZEN in mice through its antioxidant effects. Long et al. (13) reported that selenium yeast was effective in ameliorating the effects of ZEN-induced acute toxicity in mice owing to its antioxidant effects. Long et al. (14) also found that selenium yeast protected reproductive system against damage induced by ZEN by improving the murine antioxidant capacity and inhibiting reproductive cell apoptosis. Furthermore, Zhang et al. (15) reported that selenium protected against the kidney damage in mice induced by ZEN through the same mechanisms. In addition, Xiao et al. (16) found that selenium protected against ZEN-induced oxidative stress and apoptosis in chicken spleen lymphocytes by blocking reactive oxygen species generation, increasing the antioxidant capacity and reversing apoptosis. Similarly, Our results indicated that SC could alleviate the damage to the antioxidant functions of liver and blood induced by ZEN in mice. SC has excellent bioavailability and biological activity, especially antioxidant defense, compared with other organic forms of selenium in animals (17). SC not only plays the antioxidant role of organic selenium but also that of chitosan. In brief, SC has dual antioxidant effects. Both chitosan (21, 22) and selenium (23, 24) can help animal resist oxidative damage and improve the antioxidant functions. Therefore, the SC alleviation of the decrease in antioxidant functions induced by ZEN may result from the joint actions of organic selenium and chitosan.

Cytokine (e.g., IL-1β, IL-17A, IL-2 and IL-6) contents and Con A-stimulated T-cell proliferation are commonly used to evaluate immune function (25). Therefore, we tested mitogen-induced splenocyte proliferation, plasma IL concentrations and liver IL mRNA expression levels to observe the effects of SC on alleviating ZEN-induced immune function damage in mice.

To understand the mode of action of SC on immune function damage induced by ZEN in mice, we investigated its effects on T cells *in vitro* using Con A-induced splenocyte activation assays. Compared with the control group, Con A-induced splenocyte proliferation in the ZEN group decreased. Compared with the ZEN group, Con A-induced splenocyte proliferation in the ZEN+Se1, ZEN+Se2 and ZEN+Se3 groups increased (Table 2). The results indicated that ZEN had direct inhibitory effects on T-cell activation and proliferation in mice, and SC could reverse these inhibitory effects. This may be explained by the following: (a) selenium can affect the calcium flux induced by T-cell receptor involvement, modulate the redox state during T-cell activation, and also play an important role in T-cell proliferation and differentiation (26); and (b) selenium mediates T-cell immunity through an antioxidant mechanism (27).

To better understand the mode of action of SC on reduced cell-mediated immune functions induced by ZEN in mice, we investigated its effects on regulating cytokine (IL) expression and secretion levels. Compared with the control group, IL-1β, IL-17A, IL-2 and IL-6 contents and mRNA expression levels in the ZEN group decreased. Compared with the ZEN group, IL-1β, IL-17A, IL-2 and IL-6 contents and mRNA expression levels in the ZEN+Se2 and ZEN+Se3 groups increased. In addition, compared with the ZEN group, IL-1β and IL-6 contents and IL-2 and IL-17A mRNA expression levels in the ZEN+Se1 group also increased (Figures 3, 4). The results indicated that ZEN caused immunosuppression in mice, which was manifested by reduced cytokine (IL) expression and secretion levels; and SC could reverse the immunosuppression induced by ZEN.

The present study showed that ZEN supplementation led to decreases in the IL-1β, IL-17A, IL-2 and IL-6 contents and mRNA expression levels in mice. Similarly, Lee et al. (28) reported that ZEN might decrease innate immunity by attenuating the production of proinflammatory mediators and decreasing the secretion of TNF-α, IL-1β, IL-6 and other proinflammatory cytokines. Pistol et al. (29) also revealed that ZEN decreased the levels of IL-1β, IL-6, and other cytokines in the livers of the experimentally intoxicated piglets. In addition, Yang et al. (30) reported that feed supplementation with 2.0 mg/kg ZEN or more decreases IL-2 levels in female piglets. However, another study showed that dietary ZEN at the levels of 300 μg/kg could increase IL-2 contents in Gilts (31). This discrepancy may be explained by the effect of ZEN on immune function being dose dependent. A low dose of ZEN stimulates immune function, while a high dose of ZEN suppresses immune function. Therefore, the effects of ZEN on IL contents and mRNA expression levels also depends on the dose. The higher ZEN dose resulted in inhibitory effects on IL contents and mRNA expression levels in the present study, whereas a lower ZEN dose in Shen et al. (31) had a stimulatory effect on IL contents.

Our results also showed that SC supplementation led to increased IL-1β, IL-17A, IL-2 and IL-6 contents and mRNA expression levels induced by ZEN in mice, which indicated that SC could reverse the ZEN-induced immunosuppression. Both organic selenium (32, 33) and chitosan (34, 35) can regulate the immune functions of animals. Furthermore, SC can regulate the immune functions of mice (17) and breeder roosters (36). Moreover, Abdel-Tawwab et al. (37) reported that dietary supplementation with chitosan significantly reverses immunosuppression and transcriptomic responses induced by ZEN in fish. Thus, we hypothesized that SC can reverse the immunosuppression induced by ZEN, similar to the simultaneous actions of organic selenium and chitosan. Additionally, the mechanism may be that the increase in IL-1β, IL-17A, IL-2 and IL-6 messenger RNA by SC increases their secretion levels, thereby improving mouse immunity and alleviating ZEN-induced immunosuppression. However, the specific mechanism behind SC's reversal of immunosuppression induced by ZEN needs to be explored further.

Here, dietary supplementation of 0.4 and 0.6 mg/kg SC (calculated as selenium) had a modulating effect on almost all the measured indicators in mice, whereas dietary supplementation of 0.2 mg/kg SC (calculated as selenium) only had a regulatory effect on some of the measured indicators. Therefore, considering the cost and effects, the optimal dose of CS for resistance to ZEN in mice was 0.4 mg/kg.

## Conclusions

ZEN negatively affected antioxidant functions and immunosuppression in mice, but these effects could be alleviated by SC.

## Data availability statement

The original contributions presented in the study are included in the article/supplementary material, further inquiries can be directed to the corresponding author.

## Ethics statement

The animal study was reviewed and approved by Animal Care and Use Committee of Tianjin Agricultural University.

## Author contributions

SQ: investigation, data analysis, validation, and writing—original draft and editing. FS: investigation, data analysis, and writing—original draft. FZ: investigation, validation, and resources. LL: supervision, project administration, and funding acquisition. FC: writing—review and editing, supervision, and project administration. All authors contributed to the article and approved the submitted version.

## Funding

This research was supported by Key Project of Tianjin Natural Science Foundation (20JCZDJC00170), Shandong Natural Science Foundation (ZR2021MC150), Shandong Science and Technology Small and Medium Enterprises Innovation Ability Improvement Project (2021tsgc1303), and Shandong Modern Agricultural Technology and Industry System, China (SDAIT-11-07).

## Conflict of interest

The authors declare that the research was conducted in the absence of any commercial or financial relationships that could be construed as a potential conflict of interest.

## Publisher's note

All claims expressed in this article are solely those of the authors and do not necessarily represent those of their affiliated organizations, or those of the publisher, the editors and the reviewers. Any product that may be evaluated in this article, or claim that may be made by its manufacturer, is not guaranteed or endorsed by the publisher.
